# Towards accurate exclusion of neonatal bacterial meningitis: a feasibility study of a novel 16S rDNA PCR assay

**DOI:** 10.1186/s12879-020-05160-x

**Published:** 2020-06-22

**Authors:** Arthur Abelian, Thomas Mund, Martin D. Curran, Stuart A. Savill, Nipa Mitra, Carol Charan, Amanda L. Ogilvy-Stuart, Hugh R. B. Pelham, Paul H. Dear

**Affiliations:** 1 Department of Paediatrics, Maelor Hospital, Betsi Cadwaladr University LHB, 12 Fleming Drive, Wrexham, LL11 2BP UK; 2 MRC Laboratory of Molecular Biology, Cambridge, UK; 3 Clinical Microbiology, Public Health England, Addenbrookes Hospital, Cambridge, UK; 4 North Wales Clinical Research Centre, Betsi Cadwaladr University LHB, Wrexham, UK; 5 Rosie Hospital, Cambridge, UK; 6 Mote Research Ltd., Babraham, Cambridge, UK

**Keywords:** Neonatal bacterial meningitis, Broad-range PCR, Ethidium monoazide

## Abstract

**Background:**

PCRctic is an innovative assay based on 16S rDNA PCR technology that has been designed to detect a single intact bacterium in a specimen of cerebro-spinal fluid (CSF). The assay’s potential for accurate, fast and inexpensive discrimination of bacteria-free CSF makes it an ideal adjunct for confident exclusion of bacterial meningitis in newborn babies where the negative predictive value of bacterial culture is poor. This study aimed to stress-test and optimize PCRctic in the “field conditions” to attain a clinically useful level of specificity.

**Methods:**

The specificity of PCRctic was evaluated in CSF obtained from newborn babies investigated for meningitis on a tertiary neonatal unit. Following an interim analysis, the method of skin antisepsis was changed to increase bactericidal effect, and snap-top tubes (Eppendorf™) replaced standard universal containers for collection of CSF to reduce environmental contamination.

**Results:**

The assay’s specificity was 90.5% in CSF collected into the snap-top tubes – up from 60% in CSF in the universal containers. The method of skin antisepsis had no effect on the specificity. All CSF cultures were negative and no clinical cases of neonatal bacterial meningitis occurred during the study.

**Conclusions:**

A simple and inexpensive optimization of CSF collection resulted in a high specificity output. The low prevalence of neonatal bacterial meningitis means that a large multi-centre study will be required to validate the assay’s sensitivity and its negative predictive value.

## Background

The clinical signs of neonatal meningitis are very non-specific [1] and the current UK practice is to test cerebro-spinal fluid (CSF) for meningitis in all newborn babies with suspected sepsis and raised C-reactive protein [2]. With many babies exposed to antibiotics intrapartum [3] and with nearly all babies receiving antibiotics before the lumbar puncture [2], a negative result in CSF bacterial culture does not rule out bacterial meningitis [4–6]. CSF pleocytosis can be indicative of meningitis, but CSF microscopy can be difficult to interpret: (i) up to 50% of neonatal lumbar punctures result in blood-stained CSF [7, 8], (ii) there is uncertainty as to what constitutes a normal CSF white cell count in healthy babies [9, 10], and (iii) “normal” CSF microscopy does not exclude bacterial meningitis [11]. Even when blood cultures are positive, the bacteria in the blood do not necessarily match those in CSF [11].

Based on clinical and microbiological data [12–14], the rate of neonatal bacterial meningitis in the UK is low (0.2 to 0.3 per 1000 live births). This notwithstanding, 20–40 newborn babies per 1000 live births are investigated for meningitis [15]. The overwhelming majority of these babies do not have bacterial meningitis [6, 15, 16]. However, poor sensitivity of CSF bacterial culture and uncertainties in the interpretation of CSF microcopy can result in unnecessary hospitalization and treatment, with associated costs and risks [17]. We therefore speculated that an assay sensitive enough to accurately discriminate bacteria-free CSF would facilitate accurate exclusion of bacterial meningitis, with early discontinuation of antibiotic treatment and discharge from hospital. To that end, we developed PCRctic – an assay based on the broad range 16S rDNA PCR technology, enhanced to detect a single bacterium in a standard neonatal CSF specimen (200 μl).

A recent Cochrane review highlighted limitations of the reported molecular assays for neonatal sepsis [18]. Multiplex PCR assays, which simultaneously test for a number of organisms, on the whole showed poor sensitivity and specificity for neonatal sepsis (76 and 81%, respectively; based on six reports). The performance of multiplex PCR for neonatal meningitis remains to be addressed [19]. Standard broad-range 16S rDNA PCR assays fared better but rely on high concentration of bacteria in the samples (10^2^–10^3^ colony-forming units per milliliter [CFU/ml]) [20] and are therefore unlikely to have adequate sensitivity for accurate exclusion of meningitis.

The sensitivity of standard 16S rDNA PCR is limited due to the universal detection of free bacterial DNA contaminating samples and reagents [21]. To address this limitation, PCRctic utilizes ethidium bromide monoazide – a photo-reactive DNA-binding agent [22] – to eliminate the unwanted free bacterial DNA. This then allows the sensitivity to be boosted by employing a nested PCR format to selectively detect any intact bacteria present in the specimen.

The broad range and high sensitivity of the assay promise an unprecedented scope for the evaluation of the bacterial carriage in clinical specimens but also make it vulnerable to false positive results due to the ubiquitous environmental bacteria. This feasibility study of PCRctic was conducted to establish the operating procedures that result in a clinically useful level of specificity.

## Methods

### Study participants and oversight

From October 2016 to September 2017 we recruited newborn babies of at least 34 weeks post-menstrual age undergoing lumbar puncture to exclude meningitis on either the postnatal wards or on the neonatal unit at the Rosie Hospital, Cambridge University Hospitals NHS Trust. Informed consent was obtained before CSF was analysed by PCRctic. UK National Research Ethics Committee and UK Health Research Authority (HRA) approved the study.

### Study interventions

All babies recruited into the study had their CSF tested by bacteriological culture and in PCRctic. The study had two phases. In the first, at least five drops of CSF were collected into each of three sterile universal containers (ISS Ltd., UK). CSF in two of these was tested using the standard laboratory assessment for meningitis, and, following parental consent, CSF in the third container was tested in PCRctic in the clinical microbiology laboratory, Addenbrookes Hospital, Cambridge University Hospitals NHS Trust. Whilst the 30 ml sterile universal containers are routinely used across the UK for CSF collection, special care is needed to avoid contamination through the handling of the screw-on tops. In the second phase, CSF for PCRctic was collected into 1.5 ml sterile individually sealed snap-top tubes (Eppendorf Biopur, Eppendorf AG, Germany). Lumbar puncture technique was as standard. Unisept (Molnlycke Healthcare Ltd., UK) solution of 0.05% chlorhexidine was used in the first phase of the study for skin antisepsis. This was changed to ChloraPrep (BD, UK), containing 2% chlorhexidine and 70% alcohol for the second phase of the study.

### Interim analysis

Results from the initial 39 samples suggested possible environmental contamination and the study protocol was amended to include individually sealed sterile snap-top tubes and ChloraPrep skin antisepsis (as above). The amendment was approved by HRA.

### Study outcome

The primary outcome measure was the rate of false positive results. As this was a feasibility study, the results had no effect on patient care.

### Modified 16S rDNA PCR assay (PCRctic)

**Fig. 1:**
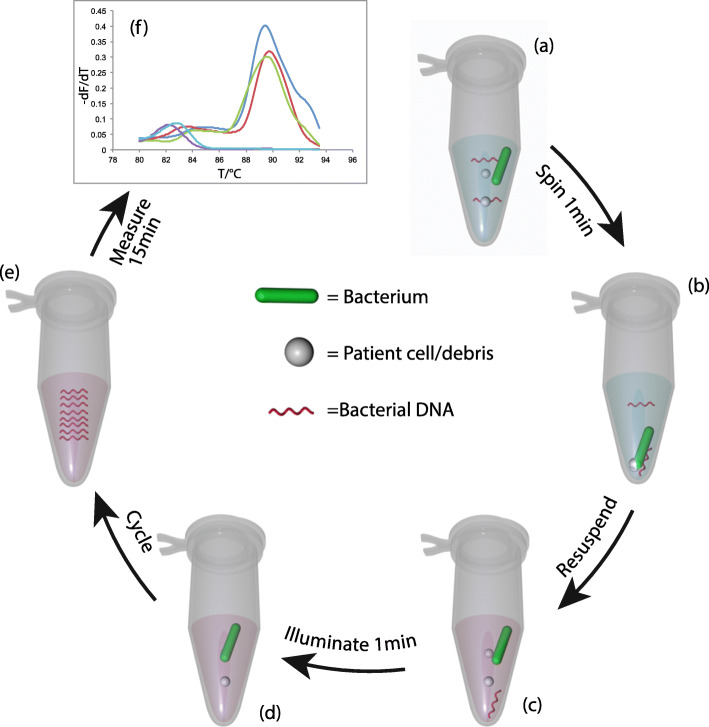
PCRctic: **a** CSF samples; **b** Intact bacteria are pelleted along with patient cells and debris; **c** The pellet is resuspended in the assay mixture (PCR reagents, ethidium azide, buffer); **d** light activates the ethidium azide, destroying exposed DNA (from dead bacteria or from contaminants in the reagents) but leaving intact bacteria unaffected; during PCR **e**, heat causes the bacteria to lyse, making their DNA available for amplification. **f** amplification products are then detected by a simple fluorometric assay (melting-curve analysis) using a widely-available real-time PCR machine. Total assay time is approximately 2 h

PCRctic (Fig. 1) uses primers against the conserved regions of bacterial ribosomal DNA. Its sensitivity and specificity derive from a single-step, closed-tube nested PCR format employing external primers (25mers) with high Tm (≈75 °C) at 50 nM (30 cycles) [333F25 CCAGACTCCTACGGGAGGCAGCAGT, 929R25 CCACATGCTCCACCGCTTGTGCGGG] and internal primers (19mers) with a low Tm (≈50 °C) at 0.25 μM (40 cycles) [800F19 TAGTCCACGCCGTAAACGA, 907R19 CCGTCAATTCATTTGAGTT]. Primers were designed against the conserved regions of the bacterial 16S rDNA gene. Briefly, 21,397 rDNA gene sequences were downloaded and aligned, and a simple script was used to identify conserved portions. From these portions, primers were designed to have a Tm (calculated as 2x[A + T] + 4x[G + C]) of 60–70 °C (outer primers) or 48–52 °C (inner primers).

**Fig. 2:**
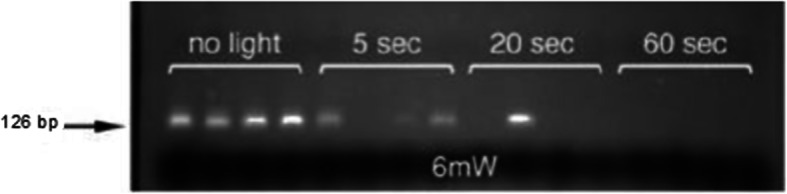
Elimination of false positives due to contaminating bacterial DNA, by photoactivation of EtA. A band at 126 bp indicates a positive result. Without photoactivation (“no light”) all specimens are positive. Exposure to light for 60 s completely eliminated false positive signal. Electrophoresis of PCRctic products in 3% agarose gel

Contamination from free bacterial DNA was eliminated by the use of ethidium bromide monoazide (EtA) [22]. Exposure of the reaction tube to light (530 nm) for 1 min causes EtA to react covalently with DNA, rendering it non-amplifiable (Fig. 2). The same illumination also destroys any residual EtA. Importantly, EtA does not penetrate intact bacteria and can therefore be used in the presence of the target (intact) bacteria before these are lysed by heat in the first PCR cycle. Typically, 180 μl of CSF were transferred into 0.2 ml PCR tubes and pelleted in a microfuge (Eppendorf: 5424, Rotor: FA-45-24-11) for 2 min at 20.000 *g*. After carefully removing the supernatant with a sterilized gel-loading tip, 10 μl of a previously-frozen PCR mastermix (80 μl KOD 10x buffer, 80 μl 10x dNTPs, 64 μl 25 mM MgSO_4_, 8 μl KOD HotStart enzyme, 40 μl 20x primer mix, 510 μl HPLC-grade water, 16 μl 10x SYBR Green, 1.6 μl 25 μM ROX, 1.25 μl 2.4 mM EtA) were added. The closed PCR tubes were then illuminated as above to photoactivate EtA. The samples were then amplified on a quantitative PCR (qPCR) system (Applied Biosytems ViiA 7; 95 °C × 3 min, then 30 cycles of: 94 °C x 10s, 70 °C x 20s, 72 °C x 30s; then 40 cycles of 94 °C x 10s, 50 °C x 20s, 72 °C x 30s) followed by a melting curve analysis.

**Fig. 3:**
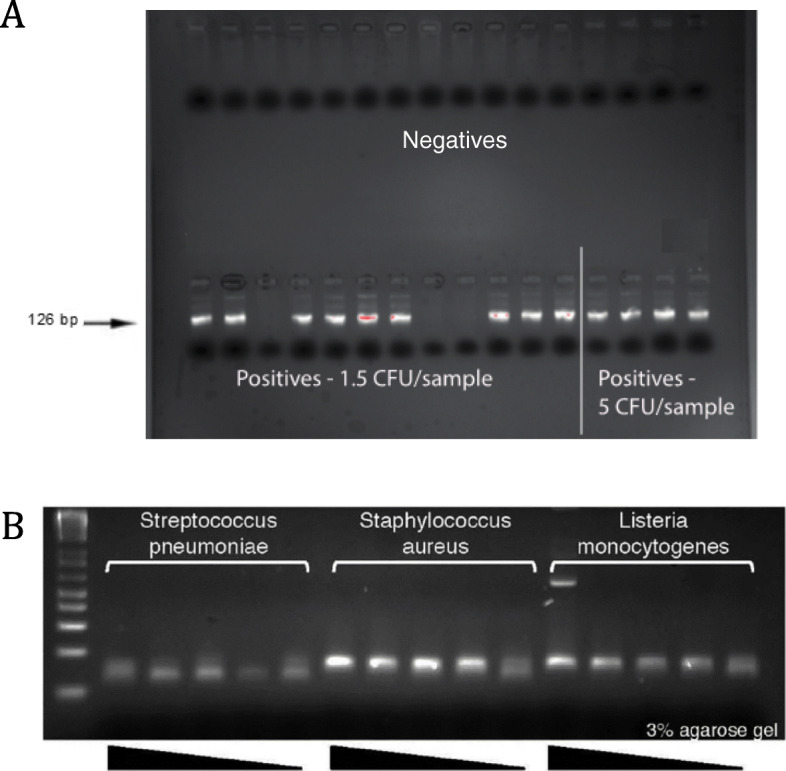
Sensitivity of PCRctic with 200-μl CSF samples “spiked” with bacteria. Electrophoresis of PCRctic products in 3% agarose gel. **a***E. coli* (DH5-alpha strain) were titrated to an average of 1.5 or 5 CFU/sample. Occasional negative results at 1.5 CFU/sample can be accounted for by the random nature of titration at this level: with an average of 1.5 CFU/sample, approximately 22% of samples should be negative. **b***Streptococcus pneumoniae*, *Staphylococcus aureus* and *Listeria monocytogenes* (patient-derived isolates) were titrated by 10-fold dilutions to ≤5 CFU/sample

**Fig. 4:**
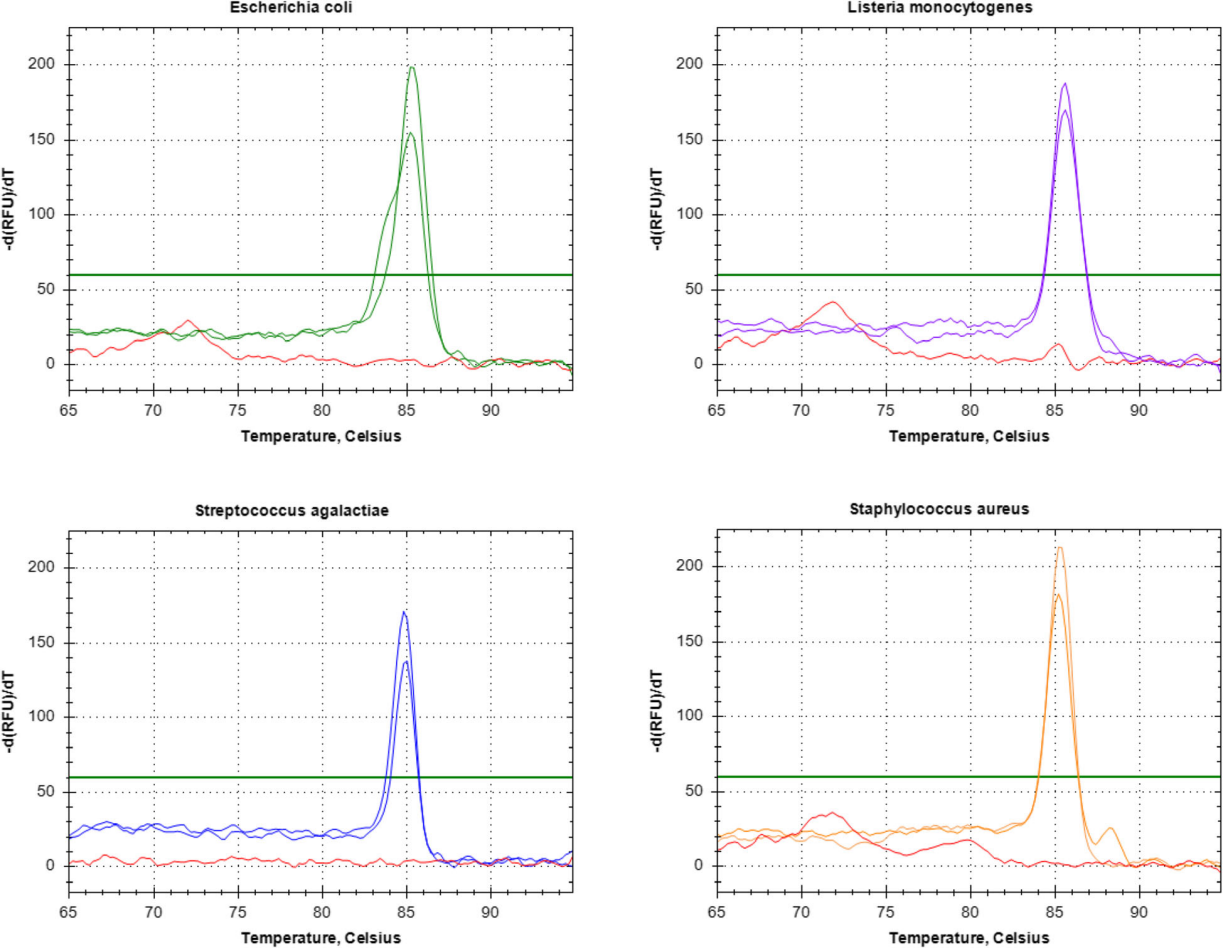
Sensitivity of PCRctic with 100-μl samples “spiked” with 10 and 5 CFUs of bacteria per reaction. Melting curve analysis of PCRctic results

Negative controls tested the mastermix in empty tubes, positive controls used spiked mastermix under the same experimental conditions (all done using the same hood, equipment and environment). Bacterial strains used were *E. coli* (DH5-alpha and ATCC 25922), *S. aureus* (ATCC 29213), *S. agalactiae* (NCTC 8181) or *L. monocytogenes (NCTC 7973)*. In CSF samples spiked with bacteria, PCRctic reliably detected as few as 1.5 CFUs (Fig. 3a&b and Fig. 4). Positives gave a single melt at 86–90 °C, in negatives primer dimers gave a single (72–75 °C) or double peak between 72 and 82 °C (not shown), which served as an internal control. For confirmation, the samples were run on 3% agarose minigels detecting the expected band of about 126 bp (size varied slightly depending on bacterial species). Positives were purified from gels and sequenced. Where possible, mixed samples were re-cloned into pBluescript vector (Agilent Technologies, USA) and individually sequenced.

### Statistical analysis

A nonparametric Mann-Whitney U test was used for the significance of the difference between the studied groups. Bayesian statistics were used to estimate the significance of the positive results under different prevalence, specificity, and sensitivity conditions.

## Results

In total, CSF from 73 babies was tested in PCRctic in parallel with standard microbiological testing. All babies received antibiotics before lumbar puncture. All bacterial cultures (blood and CSF) were negative, and all babies, including those with CSF white cell count of ≥20 per microliter (Table 1), had no neurological signs suggestive of meningitis and no baby was treated for bacterial meningitis.

**Table 1:** CSF microscopy, culture and PCRctic results in universal containers (samples 1–52) and snap-top tubes (samples 53–73)

		Microscopy			Bacteriology	
	N^o^	WCC^a^	RCC^b^	R/W^c^	Culture	PCRctic
Universal container	1	0	0		no growth	negative
	2	10	662		no growth	negative
	3	26	30,780	1183	no growth	Armatimonadetes
	4	12	6930		no growth	negative
	5	6	32,310		no growth	negative
	6	?	?		no growth	negative
	7	0	9180		no growth	negative
	8	0	260		no growth	Flavobacteriaceae [23] & Rhodobacteraceae
	9	0	6570		no growth	Hymenobacter (Flavobacteriaceae)
	10	0	0		no growth	negative
	11	24	51,120	2130	no growth	Flavobacteriaceae
	12	6	280		no growth	Ochrobactrum sp. [24]
	13	8	54,000		no growth	Mixed sequence
	14	0	137		no growth	Oscillatoriales
	15	0	690		no growth	negative
	16	8	12		no growth	Acinetobacter [25]
	17	24	20	0.8	no growth	negative
	18	22	42	1.9	no growth	negative
	19	2	4680		no growth	negative
	20	14	15,840		no growth	negative
	21	0	24		no growth	negative
	22	6	52		no growth	Sphingomonadaceae [26]
	23	8	14		no growth	Aerococcus christensenii [27] & Sneathia amnii [28]
	24	12	228		no growth	negative
	25	0	2610		no growth	negative
	26	0	140		no growth	negative
	27	10	300		no growth	negative
	28	30	11,430	381	no growth	Sneathia amnii
	29	0	3310		no growth	negative
	30	0	52		no growth	Streptococcaceae [29–31]
	31	0	2		no growth	negative
	32	4	146		no growth	negative
	33	16	22,860		no growth	Pseudomonas sp. [12–14]
	34	0	9720		no growth	Bdellovibrio
	35	0	10,440		no growth	Staphylococcus [12–14]
	36	8	49,500		no growth	negative
	37	8	1000		no growth	Ureaplasma [32]
	38	0	790		no growth	negative
	39	0	1800		no growth	negative
	40	0	40		no growth	negative
	41	0	80		no growth	negative
	42	0	24,800		no growth	negative
	43	4	1126		no growth	negative
	44	0	38		no growth	negative
	45	10	7560		no growth	Cloacibacterium rupense
	46	8	9360		no growth	negative
	47	4	284		no growth	Mixed sequence
	48	0	18,900		no growth	negative
	49	0	0		no growth	negative
	50	0	6		no growth	Methylobacterium sp.
	51	4	10,170		no growth	Pedobacter suwonensis
	52	0	0		no growth	*Staphylococcus aureus*
Snap-top tube (“Eppendorf”)	53	16	22,860		no growth	negative
	54	0	40		no growth	negative
	55	2	70		no growth	negative
	56	0	3420		no growth	negative
	57	0	0		no growth	negative
	58	2	2088		no growth	negative
	59	42	21,690	516	no growth	negative
	60	0	1080		no growth	negative
	61	0	40		no growth	negative
	62	0	132		no growth	negative
	63	12	22		no growth	Geobacter
	64	4	16		no growth	negative
	65	10	1940		no growth	negative
	66	8	4		no growth	negative
	67	4	36		no growth	Mixed sequence
	68	0	2		no growth	negative
	69	0	8820		no growth	negative
	70	0	8010		no growth	negative
	71	6	4		no growth	negative
	72	51	406,800	7976	no growth	negative
	73	6	12		no growth	negative

^a^ WCC – CSF White blood cell count, per microlitre

^b^ RCC – CSF red blood cell count, per microlitre

^c^ R/W – the ratio of CSF red to white cells (calculated only for samples with ≥20 white cells in microliter [10]. The ratio that allows accurate exclusion of neonatal bacterial meningitis remains uncertain [8]

**Fig. 5:**
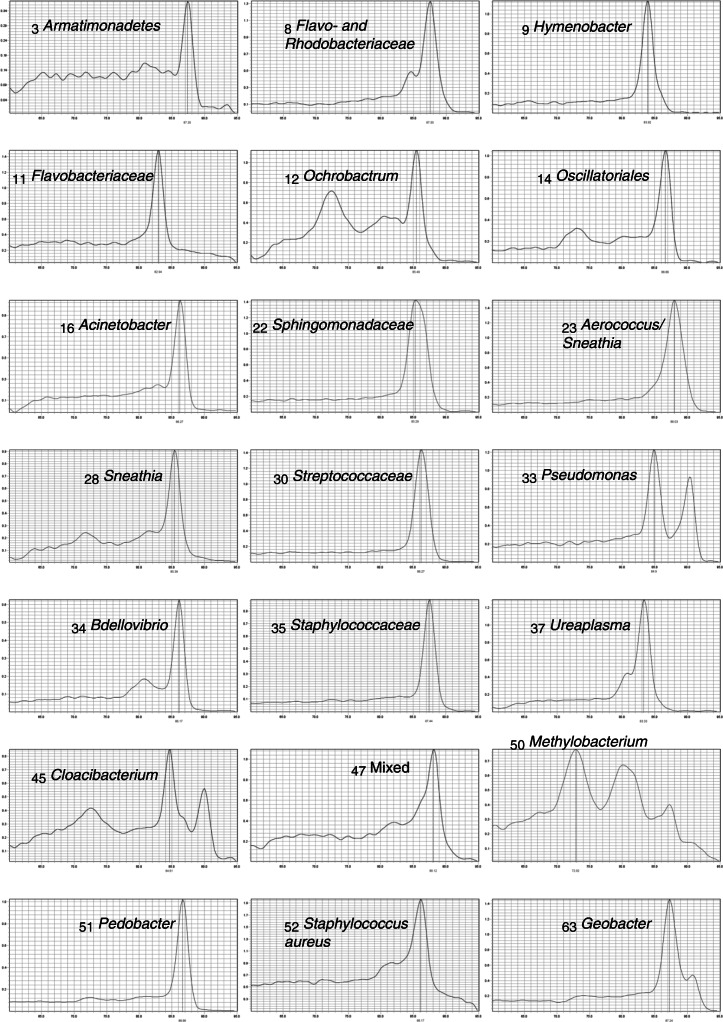
Melting curve analysis of PCRctic results obtained from clinical specimens

The first 39 samples were collected into universal containers and were from babies where Unisept was used for skin antisepsis. Sixteen of these were positive in PCRctic (41%). Sequencing revealed a diverse group of Gram-positive and Gram-negative microorganisms, some of which belonged to bacterial families associated with neonatal meningitis (Table 1, samples 1–39; Fig. 5).

At this point, considering the diversity of the bacteria detected by PCRctic, we questioned whether these originated from the CSF or were environmental contaminants acquired during lumbar puncture. We identified two possible “weak points” which might lead to environmental contamination. One was the manner of CSF collection. The universal containers may become contaminated when their screw-on tops are left on non-sterile surface at cot side during CSF collection. These were therefore replaced with individually sealed sterile snap-top Eppendorf tubes. The second “weak point” was skin antisepsis. Containing only 0.05% chlorhexidine, Unisept may have been bacteriostatic rather than bactericidal and was replaced with ChloraPrep (2% chlorhexidine and 70% alcohol) – this change was serendipitous as it reflected the comprehensive change of the unit’s practice for all procedures requiring aseptic technique.

Skin antisepsis with ChloraPrep but using universal containers (samples 40–52, Table 1) resulted in five PCRctic positive samples (5/13; 38%). Skin antisepsis with ChloraPrep and collection of CSF into snap-top Eppendorf tubes (samples 53–73, Table 1) resulted in two PCRctic positive samples (2/21; 9.5%): a Geobacter (an environmental bacterium) and a mixed species (Fig. 5). The difference between the rates of positive results in the specimens collected into the universal containers (samples 1–52) and those in the snap-top tubes (samples 53–73) was statistically significant in the Mann-Whitney U test (U = 377.5, *P* = 0.04 two-tailed). Of the 23 negative controls only one was positive in PCRctic (4%; sequencing revealed Bacillus). There was no statistically significant relationship between PCRctic result and the number of CSF white cells (U = 384.5, *P* = 0.15 two-tailed) or red cells (U = 361.5, *P* = 0.32 two-tailed).

## Discussion

Neonatal bacterial meningitis is a devastating illness with high mortality and residual neurodisability in the survivors [33–35]. Early diagnosis improves the outcomes [36]. In older children, accurate diagnosis of bacterial meningitis rests on the detection of bacteria in the CSF of a patient with clinically suspected meningitis (high temperature, headache, neck stiffness, photophobia, seizures). Accurate diagnosis of neonatal bacterial meningitis or lack thereof is more difficult for two reasons: (i) newborn babies do not develop stiff neck, photophobia or other clinical signs indicative of meningitis in older age groups [35], (ii) the sensitivity of the detection method (bacterial culture) is poor. For example, the NeonIn network reported only 41 positive CSF cultures in the period from 2005 to 2014 [37]: a rate of around 0.1 per 1000 live births, which possibly underestimates the incidence by as much as 50% [12–14]. Similarly, in a recent large multicenter study in Ireland, bacterial CSF culture missed seven cases of definite Group B Streptococcal meningitis out of 12 [6]. For these reasons, in many babies bacterial meningitis cannot be promptly confirmed or ruled out with confidence. Consequently, there is a risk of over-treatment with unnecessary hospitalisation and antibiotic exposure – a frequent problem encountered in the management of paediatric meningitis [17].

The sensitivity of the detection can be increased many-fold by PCR, which has already been widely used for diagnosis of infections (including meningitis) [38]. PCR assays can be either bacterium-specific or broad-range. Bacterium-specific PCR has not been widely used in the diagnosis of meningitis in newborn babies since it can be caused by many types of bacteria: some more common (e.g. Group B Streptococcus, *Escherichia coli*) [12–14, 35], some less so [23–32, 39–48].

In contrast, a broad-range PCR that targets the DNA region highly conserved between different types of bacteria (16S rDNA PCR), can, with few exceptions, detect bacteria of any type [21]. Until recently, the sensitivity of such broad-range PCR could not be fully harnessed due to the contamination with free bacterial DNA in the sample or in the reagents, making it inferior to bacterium-specific PCR to the order of one to two logs [21, 49].

Based on 16S rDNA PCR technology, PCRctic can detect virtually any type of bacterium. Through elimination of free bacterial DNA it can fully realize its sensitivity and detect single numbers of intact bacteria in the specimen – just as a well-designed bacterium-specific PCR can [6, 50]. These two features produce an ideal format for accurate identification of bacteria-free specimens. We hypothesized that it will have a small but clinically acceptable rate of false positive results, and a very high negative predictive value. These features can be especially useful in neonatal infection where the vast majority of tested babies do not have infection and early accurate discrimination of negative samples may assist in discontinuing or even withholding antibiotics [51, 52]. In babies with meningitis, the positive signal in PCRctic signifies the presence of bacteria with intact cellular wall. Such bacteria potentially remain viable for up to 21 days, which dictates the length of antibiotic treatment [53, 54].

Whereas skin antisepsis with either Unisept or ChloraPrep resulted in the same rate of positive results in universal containers (about 40%), the collection into snap-top tubes significantly reduced the level to 9.5%. Thus a simple and low-cost optimization of CSF handling increased the specificity of the assay from ≈60% (21 false positives out of 52 negative specimens) to 90.5% (two false positives out of 21 negative specimens). The format therefore addresses both types of environmental contamination: eliminates contaminating free bacterial DNA (ethidium azide) and reduces the risk of contamination with environmental bacteria (snap-top tubes). The performance of the negative controls (one positive out of 23) indicates that the limit of the assay’s specificity is around 96%. The assay is inexpensive: the cost of reagents is significantly less than $1, the cost of a sterile snap-top tube is $0.23. The feedback from the clinical staff was that the snap-top tubes were easy to use and no handling-related issues were reported.

Application of Bayesian analysis shows that with these false-positive rates (and assuming the assay’s sensitivity of 99% and the prevalence of bacterial meningitis of one in 150 tested babies), the chance that a positive result is truly positive (the positive predictive value) will always be less than 50% (Table 2). Thus, one important consequence of the low prevalence of neonatal meningitis is that the clinician will always have to interpret positive results cautiously, within the context of baby’s condition and other laboratory data. Another consequence is that the negative predictive value of an accurate negative result is extremely high, underscoring the advantage of such an approach for accurate exclusion of bacterial meningitis.

**Table 2:** Bayesian analysis of the positive and negative results

	Specificity (%)		
	90	96	99
Sensitivity (%)^a^	99	99	99
Prevalence^b^	1/150	1/150	1/150
Negative predictive value	0.999	0.999	0.999
Positive predictive value	0.07	0.14	0.41

^a^ assumed for the purposes of this analysis and is based on the reported sensitivity of a rigorously-designed bacterium-specific qPCR assay [6]

^b^ prevalence amongst babies tested for meningitis as based on our practice [15]

## Conclusion

In this feasibility study, an optimization of neonatal CSF collection resulted in a high specificity 16S rDNA PCR output. This study was underpowered to test the assay’s sensitivity. In fact, based on the significance level of ≤5 and 80% power, 12 cases of meningitis will be required to demonstrate a 40% improvement in sensitivity against bacterial CSF culture, and 49 cases to demonstrate a 20% improvement [55]. With the prevalence of around 0.3 per 1000 live births, the population that will need to be involved in such a cross-sectional study is between 40,000 to 160,000 live born babies. Clearly, a large multicenter study will be required. The feasibility study reported here was an important step in that direction.

## Data Availability

The datasets used and/or analysed during the current study are available from the corresponding author on reasonable request.

## Abbreviations

PCR: Polymerase chain reaction
CFU: Colony-forming unit
EtA: Ethidium monoazide bromide
KOD: Thermococcus kodakaraensis
ROX: Rhodamine X
